# Supplementary material for: Identification of shared and disease-specific host gene–microbiome associations across human diseases using multi-omic integration
Source: Nat Microbiol. 2022 May 16;7(6):780–95. doi: 10.1038/s41564-022-01121-z (PMC9159953; doi:10.1038/s41564-022-01121-z)
Supplement: Supplementary file 1 — Reporting Summary [file 41564_2022_1121_MOESM1_ESM.pdf]

nature research

Corresponding author(s): Dr. Ran Blekhman

Last updated by author(s): Mar 26, 2022

Reporting Summary

Nature Research wishes to improve the reproducibility of the work that we publish. This form provides structure for consistency and transparency in reporting. For further information on Nature Research policies, see our Editorial Policies and the Editorial Policy Checklist.

Statistics

For all statistical analyses, confirm that the following items are present in the figure legend, table legend, main text, or Methods section.

n/a	Confirmed	
☐	☒	The exact sample size (*n*) for each experimental group/condition, given as a discrete number and unit of measurement
☐	☒	A statement on whether measurements were taken from distinct samples or whether the same sample was measured repeatedly
☐	☒	The statistical test(s) used AND whether they are one- or two-sided<br>*Only common tests should be described solely by name; describe more complex techniques in the Methods section.*
☐	☒	A description of all covariates tested
☐	☒	A description of any assumptions or corrections, such as tests of normality and adjustment for multiple comparisons
☐	☒	A full description of the statistical parameters including central tendency (e.g. means) or other basic estimates (e.g. regression coefficient) AND variation (e.g. standard deviation) or associated estimates of uncertainty (e.g. confidence intervals)
☐	☒	For null hypothesis testing, the test statistic (e.g. *F*, *t*, *r*) with confidence intervals, effect sizes, degrees of freedom and *P* value noted<br>*Give P values as exact values whenever suitable.*
☒	☐	For Bayesian analysis, information on the choice of priors and Markov chain Monte Carlo settings
☒	☐	For hierarchical and complex designs, identification of the appropriate level for tests and full reporting of outcomes
☐	☒	Estimates of effect sizes (e.g. Cohen's *d*, Pearson's *r*), indicating how they were calculated

Our web collection on statistics for biologists contains articles on many of the points above.

Software and code

Policy information about availability of computer code

Data collection	No software was used in data collection.
Data analysis	All the code for analysis was written in R (version 3.3.3). The plots were generated in R using ggplot2 (version 3.2.1). Descriptions for data analyses used in the paper are described in detail in the Methods section. Code used for analyses in the paper is available at https://github.com/blekhmanlab/host_gene_microbiome_interactions. Here are the software/packages (with version number) used in our analyses:<br><br>- FastQC (version 0.11.5)<br>- Subread (version 1.4.6)<br>- biomaRt (version 2.37.4)<br>- DESeq2 (version 1.14.1)<br>- vegan (version 2.4-5)<br>- glmnet (version 2.0-13)<br>- PMA (version 1.1)<br>- hdi (version 0.1-7)<br>- stabs (version 0.6-3)<br>- ggplot2 (version 3.2.1)<br>- Cytoscape (version 3.5.1)

For manuscripts utilizing custom algorithms or software that are central to the research but not yet described in published literature, software must be made available to editors and reviewers. We strongly encourage code deposition in a community repository (e.g. GitHub). See the Nature Research guidelines for submitting code & software for further information.

Data

Policy information about availability of data

All manuscripts must include a data availability statement. This statement should provide the following information, where applicable:

- Accession codes, unique identifiers, or web links for publicly available datasets
- A list of figures that have associated raw data
- A description of any restrictions on data availability

Raw data for host RNA-seq for CRC cohort is available on the NCBI Sequence Read Archive (SRA) under BioProject ID: PRJNA816986. Raw data for previously published 16S rRNA sequencing for the CRC cohort can be accessed at PRJNA2843553 [1]. Raw data for previously published 16S rRNA sequencing and host RNA-seq for the IBD cohort can be accessed at PRJNA398089 and GSE111889, respectively [2,3]. Raw data for 16S rRNA sequencing and host RNA-seq for the IBS cohort can be accessed at PRJEB37924 and GSE146853, respectively [4]. Processed data tables for host transcriptomics and microbiome data for each disease cohort have been included as supplemental tables (Supplementary Tables S12–S17). We used the KEGG, PID, and REACTOME gene sets from MsigDB canonical pathways collection [5].

References:
1. Burns, M. B. et al. Virulence genes are a signature of the microbiome in the colorectal tumor microenvironment. Genome Med. 7, 55 (2015).
2. Lloyd-Price, J. et al. Multi-omics of the gut microbial ecosystem in inflammatory bowel diseases. Nature 569, 655–662 (2019).
3. Integrative HMP (iHMP) Research Network Consortium. The Integrative Human Microbiome Project. Nature 569, 641–648 (2019).
4. Mars, R. A. T. et al. Longitudinal Multi-omics Reveals Subset-Specific Mechanisms Underlying Irritable Bowel Syndrome. Cell 184, 1460-1473 (2020).
5. Liberzon, A. et al. Molecular signatures database (MSigDB) 3.0. Bioinformatics 27, 1739–1740 (2011).

Field-specific reporting

Please select the one below that is the best fit for your research. If you are not sure, read the appropriate sections before making your selection.

☒ Life sciences ☐ Behavioural & social sciences ☐ Ecological, evolutionary & environmental sciences

For a reference copy of the document with all sections, see nature.com/documents/nr-reporting-summary-flat.pdf

Life sciences study design

All studies must disclose on these points even when the disclosure is negative.

Sample size

We have used all publicly available data that includes both microbiome profiles and gene expression quantification from gut mucosal samples. Although no sample size calculation was performed, our results indicate that the sample size was sufficient to identify statistically significant patterns in the data.

Data exclusions

No data was excluded from the analyses.

Replication

For study reproducibility, we have made publicly available all the data and code underlying the analysis and results presented (see above and in the manuscript for the Data Availability and Code availability). Replication was only performed as part of this study examining results that are shared across disease cohorts. There is no replication of the results within each disease cohort, for the reason that for each disease cohort, only data from a single study was available, and thus replication was not possible.

Randomization

The participants were allocated within each cohort (IBD, IBS, and CRC) based on their disease status (either disease or non-disease control). We controlled for relevant covariates based on available metadata on factors that could impact gene expression or microbiome composition. Specifically, we included covariates for gender (male or female) in all three disease cohorts, disease-subtype for IBD (Crohn's Disease or ulcerative colitis), and disease-subtype for IBS (constipation (IBS-C) or diarrhea (IBS-D)).

Blinding

Since we used publicly available, previously collected data, information on blinding during data collected can be found in the respective manuscripts describing the primary datasets. In our study, blinding was not relevant since this study is not a trial and did not assess patient outcomes; since this is a hypothesis-generating study, we did not have specific expectations for results that could bias our data analysis or interpretation.

Reporting for specific materials, systems and methods

We require information from authors about some types of materials, experimental systems and methods used in many studies. Here, indicate whether each material, system or method listed is relevant to your study. If you are not sure if a list item applies to your research, read the appropriate section before selecting a response.

Materials & experimental systems

n/a	Involved in the study
☒	☐ Antibodies
☒	☐ Eukaryotic cell lines
☒	☐ Palaeontology and archaeology
☒	☐ Animals and other organisms
☐	☒ Human research participants
☒	☐ Clinical data
☒	☐ Dual use research of concern

Methods

n/a	Involved in the study
☒	☐ ChIP-seq
☒	☐ Flow cytometry
☒	☐ MRI-based neuroimaging

Human research participants

Policy information about studies involving human research participants

Population characteristics

The study did not involve any newly collected samples, only previously collected, published datasets [1-4]. The CRC cohort comprised of 44 patients with colorectal cancer, including 23 females and 21 males, with an average age of 65 years (median: 67, range: 17–91) [1]. The IBD cohort comprised of 78 individuals, including 56 individuals with IBD, and 22 individuals without IBD ("non-IBD" in HMP2) [2,3]. Out of 56 IBD patients, 34 patients had Crohn's disease (CD) and 22 patients had ulcerative colitis (UC). The individuals in this cohort included 38 females and 40 males. Age at the time of sample collection is not reported in the metadata file available for this cohort (http://ibdmdb.org). The IBS cohort is comprised of 42 individuals, including 29 individuals with IBS, and 13 healthy individuals (non-IBS) [4]. The individuals in this cohort included 31 females and 11 males, with an average age of 38 years (median: 35, range: 20–63). The original studies obtained written informed consent from study participants in each cohort.

References:
1. Burns, M. B. et al. Virulence genes are a signature of the microbiome in the colorectal tumor microenvironment. Genome Med. 7, 55 (2015).
2. Lloyd-Price, J. et al. Multi-omics of the gut microbial ecosystem in inflammatory bowel diseases. Nature 569, 655–662 (2019).
3. Integrative HMP (iHMP) Research Network Consortium. The Integrative Human Microbiome Project. Nature 569, 641–648 (2019).
4. Mars, R. A. T. et al. Longitudinal Multi-omics Reveals Subset-Specific Mechanisms Underlying Irritable Bowel Syndrome. Cell 184, 1460-1473 (2020).

Recruitment

The study did not involve any newly collected samples, only previously collected, published datasets. Information on recruitment of participants is available in the publications describing the primary samples:

- Burns, M. B. et al. Virulence genes are a signature of the microbiome in the colorectal tumor microenvironment. Genome Med. 7, 55 (2015).
- Mars, R. A. T. et al. Longitudinal Multi-omics Reveals Subset-Specific Mechanisms Underlying Irritable Bowel Syndrome. Cell 184, 1460-1473 (2020).
- Lloyd-Price, J. et al. Multi-omics of the gut microbial ecosystem in inflammatory bowel diseases. Nature 569, 655–662 (2019).
- Integrative HMP (iHMP) Research Network Consortium. The Integrative Human Microbiome Project. Nature 569, 641–648 (2019).

Ethics oversight

For the colorectal cancer cohort, all research conformed to the Helsinki Declaration and was approved by the University of Minnesota Institutional Review Board, protocol 1310E44403. For the inflammatory bowel disease and irritable bowel syndrome cohorts, ethical approval is described in their respective publications [1,2,3].

References:
1. Mars, R. A. T. et al. Longitudinal Multi-omics Reveals Subset-Specific Mechanisms Underlying Irritable Bowel Syndrome. Cell 184, 1460-1473 (2020).
2. Lloyd-Price, J. et al. Multi-omics of the gut microbial ecosystem in inflammatory bowel diseases. Nature 569, 655–662 (2019).
3. Integrative HMP (iHMP) Research Network Consortium. The Integrative Human Microbiome Project. Nature 569, 641–648 (2019).

Note that full information on the approval of the study protocol must also be provided in the manuscript.
